# Clinical factors associated with postoperative fever after bipolar TURP and GreenLight laser vaporization: a retrospective cohort study

**DOI:** 10.3389/fsurg.2026.1847729

**Published:** 2026-07-28

**Authors:** Lei Huang, Fang Guo, Can Liu, Shujun Bai, Yu Chen, Qiong Bao, Xing Luo, Jingzhen Zhu, Weihua Fu, Bishao Sun, Jiang Zhao, Jia Li

**Affiliations:** 1 Department of Urology, The Second Affiliated Hospital, Army Military Medical University, Chongqing, China; 2 State Key Laboratory of Trauma and Chemical Poisoning, Third Military Medical University (Army Medical University), Chongqing, China

**Keywords:** antimicrobial susceptibility, benign prostatic hyperplasia, bipolar transurethral resection of the prostate, GreenLight laser vaporization, postoperative fever

## Abstract

**Background:**

Postoperative fever after transurethral surgery for benign prostatic hyperplasia may prompt evaluation for infection, but interpretation is complicated by catheterization, perioperative inflammation, variable microbiological sampling, and procedure-specific differences.

**Methods:**

We retrospectively analysed 401 patients who underwent bipolar transurethral resection of the prostate (bTURP) or GreenLight laser photoselective vaporization of the prostate (GL-PVP) from October 2019 to February 2023. The primary outcome was postoperative fever recorded during the index admission. Multivariable logistic regression was used to identify associated factors. *Post hoc* model checks included assessment of multicollinearity, calibration, goodness of fit, discrimination, and five-fold internal cross-validation where feasible. Available postoperative culture and antimicrobial susceptibility records were analysed descriptively.

**Results:**

Postoperative fever was recorded in 48 of 401 patients (11.97%), with no significant difference between bTURP and GL-PVP (11.44% vs. 12.73%; *P* = 0.755). In the reported multivariable model, diabetes mellitus, a positive preoperative urine culture, preoperative lower urinary tract functional testing, preoperative indwelling catheterization, and longer operative time were associated with postoperative fever. *Post hoc* diagnostics showed no severe multicollinearity and acceptable apparent discrimination, but the limited number of events supports cautious interpretation. The supplied postoperative culture dataset contained 24 isolate records from 22 patients; Escherichia coli (11/24) and Klebsiella pneumoniae (6/24) were most frequent. Susceptibility-test denominators varied by antimicrobial agent.

**Conclusion:**

Several preoperative and procedural factors were associated with postoperative fever after bTURP or GL-PVP. These associations are hypothesis-generating and should not be interpreted as causal. Because exact fever onset, complete SIRS components, culture specimen source, and standardized perioperative antibiotic protocols were unavailable, the microbiological findings should be interpreted as exploratory local surveillance data.

## 1 Introduction

Benign prostatic hyperplasia (BPH) commonly causes bladder outlet obstruction and lower urinary tract symptoms in older men (1, 2). When medical management is insufficient, transurethral procedures include resection, vaporization, and enucleation techniques, each with different operative profiles and potentially different postoperative infectious or inflammatory risks (3–5, 18). The present cohort specifically included bTURP and GL-PVP; these procedures are distinct from endoscopic enucleation of the prostate and should not be grouped under the term enucleation.

Postoperative fever is a clinically important event after transurethral prostate surgery because it may reflect infection, procedure-related inflammation, or both (6–12). Reported rates of postoperative urinary infection vary across studies according to procedure, case mix, catheter exposure, definitions, and microbiological sampling, so single-study estimates should not be interpreted as pooled incidence. Previous studies have identified associations with bacteriuria, diabetes mellitus, catheterization, and operative characteristics, but the strength and interpretation of these associations vary across settings. We therefore evaluated factors associated with postoperative fever after bTURP and GL-PVP in a single-centre retrospective cohort and descriptively examined the available postoperative culture records. The study was designed as a local, hypothesis-generating analysis rather than an evaluation of causal mechanisms or a basis for empirical antibiotic recommendations.

## 2 Patients and methods

### 2.1 Criteria for inclusion and exclusion

We retrospectively analysed records of patients with BPH who underwent bTURP or GL-PVP at our centre from October 2019 to February 2023. Patients were excluded if they had a pre-existing systemic or severe urinary infection, previous prostatic or urethral surgery, prostate cancer, neurogenic bladder, incomplete medical records, or documented/suspected chronic prostatitis, chronic pelvic pain syndrome, acute prostatitis, or other inflammatory prostate disease at the time of surgical assessment. Chronic prostatitis/chronic pelvic pain syndrome was considered clinically when persistent pelvic pain, inflammatory symptoms, recurrent infection history, or documentation by the treating urologist was present; because this was a retrospective study, systematic NIH-CPSI scoring and prostate secretion testing were not available. After screening, 401 patients were included. Surgeon identifiers, surgeon-level case volume, learning-curve stage, and detailed case-complexity measures were not available in the supplied analytic dataset. The study was approved by the Institutional Review Board of the Second Affiliated Hospital, Army Military Medical University (Protocol 2024YD021-01) and was conducted in accordance with the Declaration of Helsinki. Written informed consent for clinical care and use of de-identified clinical data was obtained from participants or their legal representatives. The submission metadata should therefore identify this work as a retrospective human participant study.

### 2.2 Data collection and outcome definition

Collected preoperative variables included age, body mass index, hypertension, diabetes mellitus, prostate-specific antigen, free-to-total prostate-specific antigen ratio, erythrocyte sedimentation rate, prostate volume, serum creatinine, estimated glomerular filtration rate, urine leukocyte grade, preoperative urine-culture status, preoperative lower urinary tract functional testing, transrectal prostate biopsy, and indwelling catheterization. Procedure type and operative time were also recorded. Postoperative catheterization duration was available in the source worksheets but was not incorporated into the main multivariable model because it may lie on the causal pathway after surgery and had incomplete capture for one patient. The source dataset classified patients according to whether postoperative fever was recorded during the index admission. Exact onset time and the complete physiological measurements required to verify systemic inflammatory response syndrome were not preserved; therefore, the revised analysis uses postoperative fever, not SIRS, as the primary outcome. Postoperative urinary frequency, urgency, dysuria, and pain were not treated as definitive urinary tract infection criteria because catheterization and recent transurethral surgery can make these symptoms difficult to assess. The available postoperative culture worksheet contained isolate-level susceptibility records, but specimen source and collection timing were not consistently identifiable. Antibiotic-use entries did not reliably distinguish prophylaxis from treatment or provide standardized timing and dose information.

### 2.3 Statistical analysis

Analyses were performed using SPSS version 23.0. Categorical variables were compared using the Pearson chi-square test, and continuous variables were compared using independent-samples *t*-tests. No *a priori* power calculation was performed; the sample size was determined by the eligible retrospective records available during the study period. Univariate comparisons were exploratory and were not used as stand-alone evidence of causality. To address the number of comparisons in Table 1, Benjamini–Hochberg false-discovery-rate-adjusted *P* values were calculated *post hoc* as a sensitivity check. Candidate variables for multivariable logistic regression were limited according to clinical relevance, previous literature, and availability in the source dataset, with attention to the 48 outcome events. Multivariable logistic regression was used to estimate associations with postoperative fever, reported as odds ratios (ORs) with 95% confidence intervals (CIs). Missingness was reviewed before modelling; the principal analytic variables used in the regression were complete in the available dataset, whereas several clinically relevant perioperative variables were unavailable and are addressed as limitations. To examine potential multicollinearity, pairwise correlations and variance inflation factors (VIFs) were calculated. *Post hoc* model checks included apparent discrimination, Brier score, calibration slope/intercept, Hosmer–Lemeshow goodness-of-fit testing, and five-fold internal cross-validation where feasible. Reporting was reviewed against STROBE recommendations (19). A two-sided *P* value <0.05 was considered statistically significant. Culture and susceptibility results were summarised descriptively using the number tested for each antimicrobial as the denominator.

**Table 1:** Characteristics of patients with and without postoperative fever.

Indicators	Description	Postoperative fever (*n* = 48)	No postoperative fever (*n* = 353)	*P* value
Age (years)		71.02 ± 7.81	71.56 ± 8.37	0.658
Preoperative PSA (ng/mL)		11.60 ± 14.47	8.82 ± 11.38	0.207
Preoperative f-PSA/PSA		0.25 ± 0.16	0.25 ± 0.12	0.893
Erythrocyte sedimentation rate (mm/h)		15.73 ± 17.86	13.15 ± 13.90	0.340
Preoperative prostate volume (mL)		74.80 ± 28.41	56.25 ± 25.94	<0.001
Preoperative SCr (*μ*mol/L)		95.67 ± 60.27	103.24 ± 56.37	0.285
Preoperative eGFR (mL/min)		75.85 ± 19.28	72.94 ± 20.12	0.379
Operative time (min)		124.56 ± 32.34	95.74 ± 31.23	<0.001
Surgical procedure, *n* (%)	bTURP	27 (56.25)	209 (59.21)	0.755
	GL-PVP	21 (43.75)	144 (40.79)	
BMI ≥25 kg/m^2^, *n* (%)	Yes	22 (45.83)	139 (39.38)	0.434
	No	26 (54.17)	214 (60.62)	
Hypertension, *n* (%)	Yes	16 (33.33)	126 (35.69)	0.872
	No	32 (66.67)	227 (64.31)	
Diabetes mellitus, *n* (%)	Yes	11 (22.91)	24 (6.80)	<0.001
	No	37 (77.08)	329 (93.20)	
Preoperative urine leukocytes ≥3+, *n* (%)	Yes	14 (29.17)	51 (14.45)	0.019
	No	34 (70.83)	302 (85.55)	
Positive preoperative urine culture, *n* (%)	Yes	21 (43.75)	52 (14.73)	<0.001
	No	27 (56.25)	301 (85.27)	
Preoperative lower urinary tract functional testing, *n* (%)	Yes	40 (83.33)	218 (61.76)	0.003
	No	8 (16.67)	135 (38.24)	
Preoperative transrectal prostate biopsy, *n* (%)	Yes	22 (45.83)	144 (40.79)	0.534
	No	26 (54.17)	209 (59.21)	
Preoperative indwelling catheterization, *n* (%)	Yes	30 (62.50)	125 (35.41)	<0.001
	No	18 (37.50)	228 (64.59)	

Univariate comparisons are exploratory. In a *post hoc* Benjamini-Hochberg false-discovery-rate sensitivity check, prostate volume, operative time, diabetes mellitus, positive preoperative urine culture, preoperative lower urinary tract functional testing, preoperative indwelling catheterization, and urine leukocytes >=1 + remained noteworthy; adjusted *P* values should be interpreted descriptively.

## 3 Results

### 3.1 Incidence of postoperative fever

**Figure 1:**
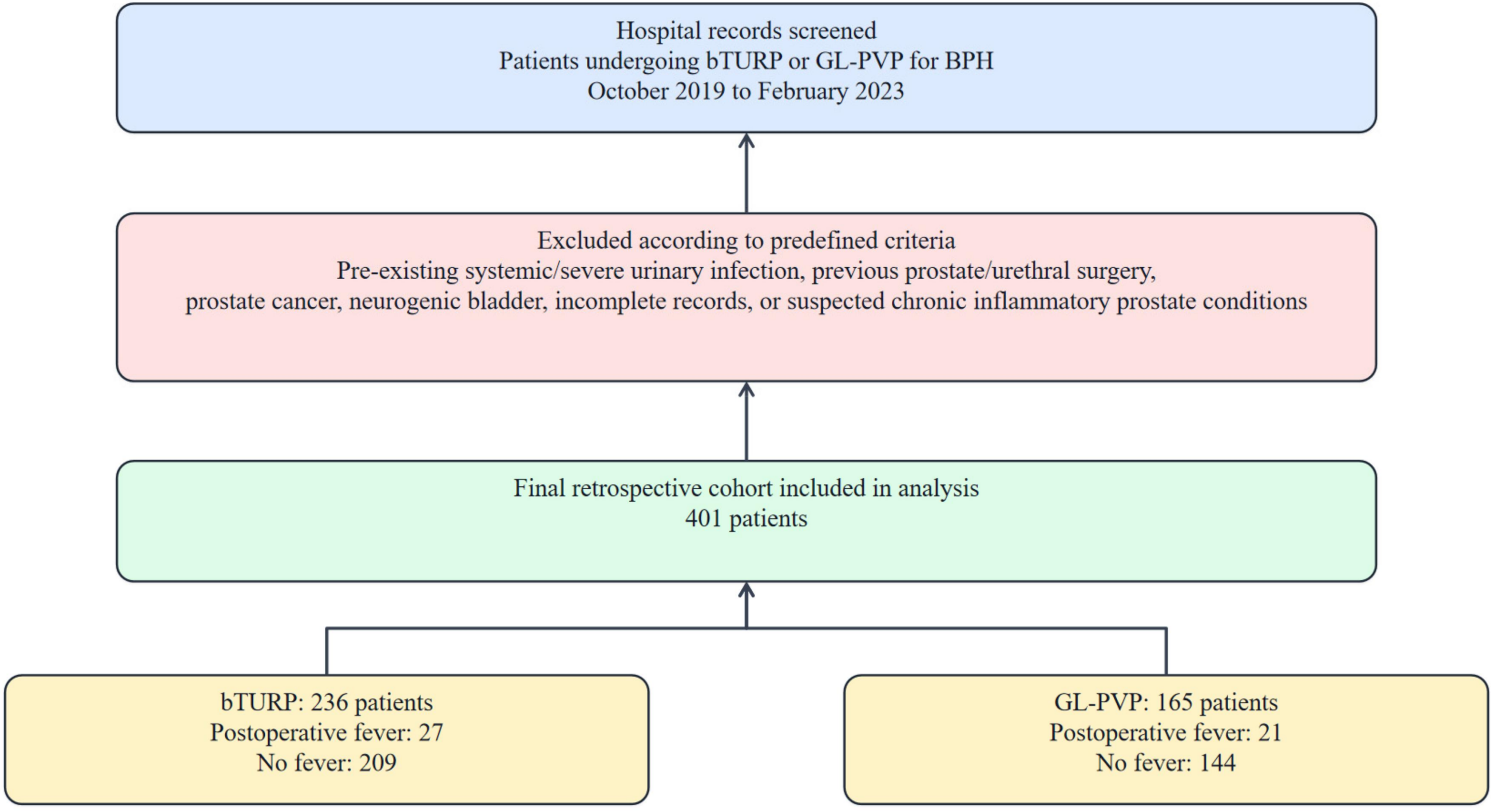
Patient selection and analysis flow.

Among 401 patients, 236 underwent bTURP and 165 underwent GL-PVP. Postoperative fever was recorded in 27 bTURP cases and 21 GL-PVP cases. The overall incidence was 11.97% (48/401), and incidence did not differ significantly between bTURP and GL-PVP (11.44% vs. 12.73%; *P* = 0.755; Table 2). A patient selection and grouping summary is shown in Figure 1.

**Table 2:** Incidence of postoperative fever.

Surgical procedure	Postoperative fever cases	Total cases	Incidence of postoperative fever	*P* value
bTURP	27	236	11.44%	/
GL-PVP	21	165	12.73%	0.755
Total operations	48	401	11.97%	/

### 3.2 Characteristics of patients with and without postoperative fever

Patients with postoperative fever had higher frequencies of diabetes mellitus (22.91% vs. 6.80%), positive preoperative urine culture (43.75% vs. 14.73%), preoperative indwelling catheterization (62.50% vs. 35.41%), and preoperative lower urinary tract functional testing (83.33% vs. 61.76%). They also had larger prostates (74.80 ± 28.41 vs. 56.25 ± 25.94 mL) and longer operative times (124.56 ± 32.34 vs. 95.74 ± 31.23 min). These unadjusted comparisons are presented in Table 1.

### 3.3 Multivariable associations with postoperative fever

**Figure 2:**
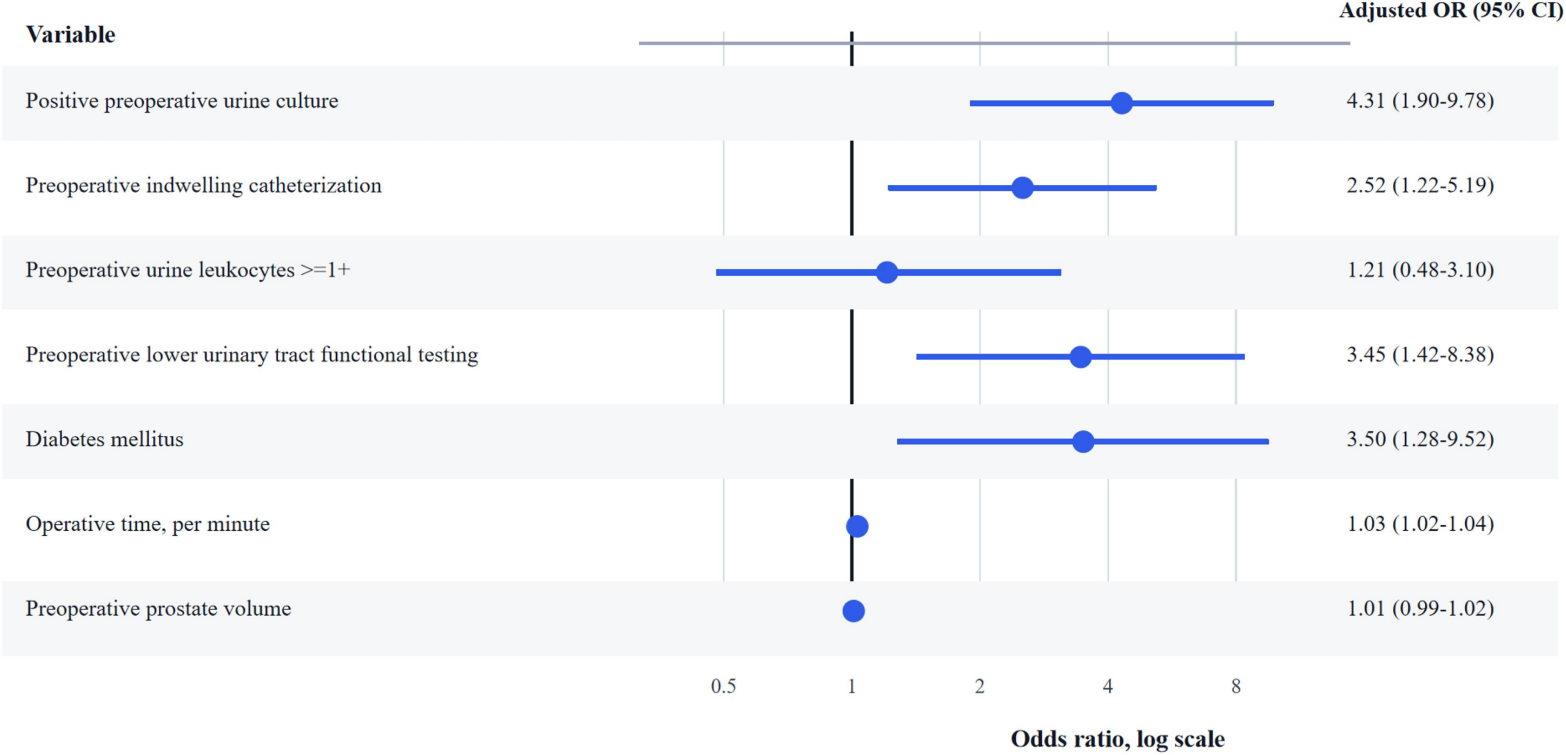
Forest plot of multivariable associations with postoperative fever.

In the reported multivariable model, positive preoperative urine culture (adjusted OR: 4.31, 95% CI: 1.90–9.78; *P* < 0.001), preoperative indwelling catheterization (OR: 2.52, 95% CI: 1.22–5.19; *P* = 0.012), preoperative lower urinary tract functional testing (OR: 3.45, 95% CI: 1.42–8.38; *P* = 0.006), diabetes mellitus (OR: 3.50, 95% CI: 1.28–9.52; *P* = 0.014), and operative time (OR: 1.03 per minute, 95% CI: 1.02–1.04; *P* < 0.001) were associated with postoperative fever (Table 3 and Figure 2). Prostate volume was not significant after adjustment. In a *post hoc* collinearity assessment, VIFs ranged from 1.015 to 1.266. Operative time and prostate volume showed a moderate correlation (*r* = 0.441), whereas the other pairwise correlations were weak. Additional *post hoc* diagnostic checks using the complete case-level variables available for model validation showed an apparent AUC of 0.824, Brier score of 0.080, Hosmer–Lemeshow chi-square of 10.37 (8 df; *P* = 0.240), calibration intercept of −0.005, calibration slope of 1.045, and five-fold cross-validated AUC of 0.807 (Table 4). These results suggest reasonable internal fit for an exploratory model but do not eliminate overfitting, especially given 48 outcome events and approximately 6.9 events per parameter in the reported seven-predictor model.

**Table 3:** Multivariable logistic regression analysis of factors associated with postoperative fever.

Variable	Regression coefficient (beta)	*P* value	Adjusted OR (95% CI)
Positive preoperative urine culture	1.46	<0.001	4.31 (1.90–9.78)
Preoperative indwelling catheterization	0.92	0.012	2.52 (1.22–5.19)
Preoperative urine leukocytes ≥3+	0.19	0.684	1.21 (0.48–3.10)
Preoperative lower urinary tract functional testing	1.24	0.006	3.45 (1.42–8.38)
Diabetes mellitus	1.25	0.014	3.50 (1.28–9.52)
Operative time, per minute	0.03	<0.001	1.03 (1.02–1.04)
Preoperative prostate volume	0.01	0.393	1.01 (0.99–1.02)

**Table 4:** *Post hoc* model diagnostic and validation checks.

Domain	Metric	Result
Sample size	Outcome events	48 of 401 patients
Overfitting	Events per parameter	8.0 in the reproducible diagnostic model; approximately 6.9 in the reported seven-predictor model
Discrimination	Apparent AUC	0.824
Discrimination	Five-fold cross-validated AUC	0.807
Calibration	Calibration intercept/slope	−0.005/1.045
Goodness of fit	Hosmer-Lemeshow test	chi-square=10.37, df = 8, *P* = 0.240
Overall prediction error	Brier score	0.080 apparent; 0.083 cross-validated

Diagnostic statistics are *post hoc* checks intended to describe internal fit of an exploratory association model, not to establish a validated prediction tool.

### 3.4 Organisms in available postoperative culture records

**Figure 3:**
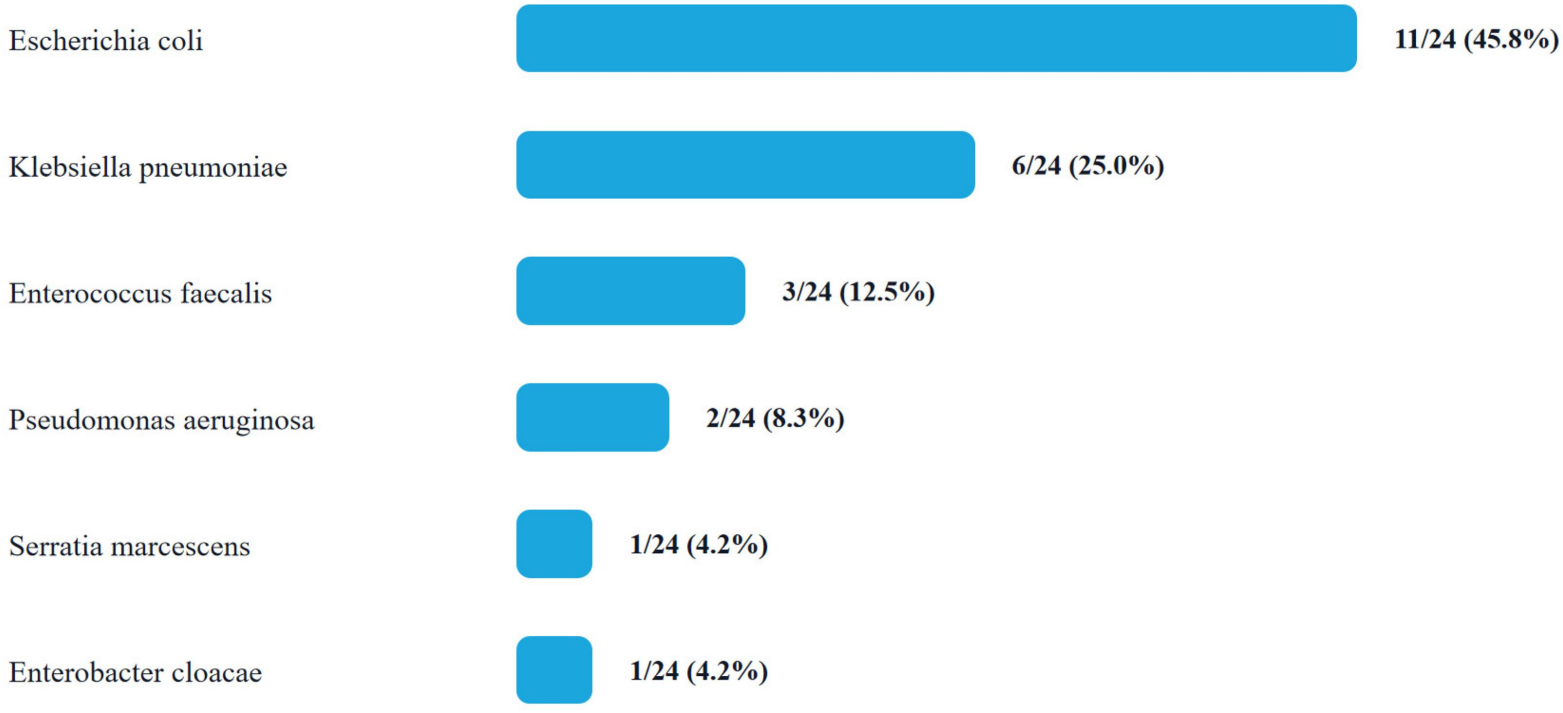
Distribution of available postoperative isolate records.

The supplied postoperative culture worksheet contained 24 isolate records from 22 patients. Escherichia coli was the most frequent organism (11/24, 45.8%), followed by Klebsiella pneumoniae (6/24, 25.0%), Enterococcus faecalis (3/24, 12.5%), Pseudomonas aeruginosa (2/24, 8.3%), Serratia marcescens (1/24, 4.2%), and Enterobacter cloacae (1/24, 4.2%; Table 5 and Figure 3). Gram-negative bacilli accounted for 21 of 24 isolate records. Because specimen source and exact collection timing were not consistently available, these isolates cannot be used to establish the cause of postoperative fever.

**Table 5:** Distribution of organisms in available postoperative culture records.

Organism	Isolate records, *n*/*n* (%)
Escherichia coli	11/24 (45.8%)
Klebsiella pneumoniae	6/24 (25.0%)
Enterococcus faecalis	3/24 (12.5%)
Pseudomonas aeruginosa	2/24 (8.3%)
Serratia marcescens	1/24 (4.2%)
Enterobacter cloacae	1/24 (4.2%)

### 3.5 Antimicrobial susceptibility results

Susceptibility testing was not performed uniformly for every isolate, so the denominator varied by antimicrobial agent (Table 6). Among agents tested in at least 20 isolates, resistance was frequent for ampicillin (21/23, 91.3%), ciprofloxacin (20/23, 87.0%), levofloxacin (20/23, 87.0%), trimethoprim-sulfamethoxazole (18/21, 85.7%), tetracycline (17/21, 81.0%), and gentamicin (16/23, 69.6%). Meropenem and polymyxin B each showed susceptibility in 20 of 21 tested isolates (95.2%). These descriptive results reflect a small, selected, single-centre isolate set and should not be used alone to select prophylactic or empirical therapy.

**Table 6:** Antimicrobial susceptibility results; denominators reflect the number of isolates tested for each agent.

Antimicrobial	Susceptible, *n*/*n* (%)	Intermediate, *n*/*n* (%)	Resistant, *n*/*n* (%)
Meropenem	20/21 (95.2%)	0/21 (0.0%)	1/21 (4.8%)
Polymyxin B	20/21 (95.2%)	0/21 (0.0%)	1/21 (4.8%)
Piperacillin-tazobactam	19/23 (82.6%)	3/23 (13.0%)	1/23 (4.3%)
Tigecycline	15/19 (78.9%)	4/19 (21.1%)	0/19 (0.0%)
Imipenem	16/23 (69.6%)	3/23 (13.0%)	4/23 (17.4%)
Cefoperazone-sulbactam	14/23 (60.9%)	7/23 (30.4%)	2/23 (8.7%)
Amikacin	11/23 (47.8%)	0/23 (0.0%)	12/23 (52.2%)
Cefepime	10/23 (43.5%)	1/23 (4.3%)	12/23 (52.2%)
Ceftazidime	8/21 (38.1%)	3/21 (14.3%)	10/21 (47.6%)
Minocycline	7/20 (35.0%)	0/20 (0.0%)	13/20 (65.0%)
Gentamicin	7/23 (30.4%)	0/23 (0.0%)	16/23 (69.6%)
Cefuroxime	5/20 (25.0%)	2/20 (10.0%)	13/20 (65.0%)
Amoxicillin-clavulanate	4/21 (19.0%)	9/21 (42.9%)	8/21 (38.1%)
Tetracycline	4/21 (19.0%)	0/21 (0.0%)	17/21 (81.0%)
Trimethoprim-sulfamethoxazole	3/21 (14.3%)	0/21 (0.0%)	18/21 (85.7%)
Ciprofloxacin	3/23 (13.0%)	0/23 (0.0%)	20/23 (87.0%)
Levofloxacin	3/23 (13.0%)	0/23 (0.0%)	20/23 (87.0%)
Ampicillin	2/23 (8.7%)	0/23 (0.0%)	21/23 (91.3%)

## 4 Discussion

This study evaluates postoperative fever after two correctly classified transurethral procedures for BPH, bTURP and GL-PVP. The principal finding is that several patient and procedural characteristics were associated with fever in the available retrospective records. These results are broadly consistent with previous reports of infectious complications after transurethral prostate surgery (6–13) and should be interpreted as local risk estimates rather than as evidence of novel causal mechanisms.

Positive preoperative urine culture, diabetes mellitus, and preoperative indwelling catheterization were associated with postoperative fever after adjustment. These associations are clinically plausible because they may identify patients with greater baseline susceptibility to infection or more intensive urinary tract instrumentation. However, the analysis cannot distinguish infection-related fever from non-infectious postoperative inflammation. The association with preoperative lower urinary tract functional testing should also be interpreted cautiously. Such testing may provide important diagnostic information in selected patients with complex lower urinary tract symptoms, detrusor dysfunction, or uncertain obstruction; therefore, the present finding should not be interpreted as evidence to avoid testing when clinically indicated. Instead, it suggests that patients undergoing preoperative instrumentation may warrant careful urine-culture review, catheter-care optimization, and perioperative monitoring. The precise testing protocol was not available, and testing may be a surrogate marker for more complex lower urinary tract dysfunction or different preoperative pathways rather than a direct cause of fever.

Longer operative time remained associated with postoperative fever, whereas prostate volume did not remain significant after adjustment. Operative time and prostate volume were moderately correlated, but the available VIFs did not indicate severe multicollinearity. Nevertheless, operative time may still represent prostate size, technical difficulty, surgeon-level practice, case complexity, energy modality, or intraoperative tissue handling. Procedure-specific effects also require caution: although bTURP and GL-PVP had similar fever incidence in this cohort, other approaches, including enucleation and technique-sparing modifications, may have different operative and postoperative profiles (18). Because surgeon identifiers, learning-curve stage, ASA classification, comorbidity indices, glycaemic control, and detailed complexity measures were unavailable, the present study cannot establish an independent causal effect of operative duration.

The culture analysis provides a descriptive view of organisms and susceptibility results in the available postoperative records. Gram-negative bacilli predominated, and resistance to fluoroquinolones and several commonly tested agents was frequent, consistent with broader concerns about antimicrobial resistance in urinary pathogens (14–17). However, the culture dataset contained only 24 isolate records from 22 patients, testing panels varied among isolates, and specimen source and collection timing were not consistently identifiable. Conventional culture also has limited sensitivity, can be affected by prior antimicrobial exposure, and may miss culture-negative infection, polymicrobial infection, or fastidious organisms. Molecular diagnostic approaches, including targeted PCR panels or metagenomic sequencing, may provide more comprehensive pathogen characterization in future prospective studies, but they were not available in this retrospective dataset. These limitations preclude attribution of fever to a urinary source and preclude recommendations for prophylactic or empirical antibiotic selection from these data alone. Treatment decisions should instead follow specimen-specific culture results, illness severity, local stewardship guidance, and current clinical guidelines (14–17).

## 5 Limitations

This study has several important limitations. First, the source dataset recorded postoperative fever but did not preserve exact onset timing or all variables required to verify SIRS, so early physiological fever could not be separated from later infectious fever. Second, the retrospective single-centre design is vulnerable to selection bias, diagnostic misclassification, and residual confounding. Although patients with documented or suspected chronic prostatitis, chronic pelvic pain syndrome, acute prostatitis, or other inflammatory prostate disease were excluded, systematic prospective inflammatory-prostate assessment was not performed. Third, the model was limited by 48 outcome events, no *a priori* power calculation, and an events-per-parameter ratio of approximately 6.9 in the reported multivariable model; overfitting therefore remains possible despite *post hoc* internal checks. Fourth, several clinically important variables were unavailable or insufficiently standardized, including perioperative antibiotic prophylaxis protocol, antibiotic timing and dosing, postoperative catheterization management, surgeon-level variability, ASA classification, overall comorbidity burden, perioperative glycaemic control, and detailed case-complexity measures. Fifth, the precise protocol for preoperative lower urinary tract functional testing was not documented, limiting causal interpretation. Sixth, postoperative urine-culture coverage, specimen source, and collection timing were not consistently identifiable, antimicrobial susceptibility testing used variable panels, and conventional culture may miss culture-negative, polymicrobial, or fastidious infections. These limitations mean that the findings should be interpreted as exploratory associations that require prospective, STROBE-compliant validation with standardized perioperative variables and microbiological sampling.

## 6 Conclusion

In this retrospective cohort, diabetes mellitus, positive preoperative urine culture, preoperative lower urinary tract functional testing, preoperative indwelling catheterization, and longer operative time were associated with postoperative fever after bTURP or GL-PVP. The findings support careful preoperative assessment and prospective validation but do not establish causality. The small, incompletely characterized culture dataset provides exploratory local surveillance only and does not support source attribution or stand-alone antibiotic recommendations.

## Data Availability

The original contributions presented in the study are included in the article/Supplementary Material, further inquiries can be directed to the corresponding authors.
